# Alzheimer’s disease: relevant molecular and physiopathological events affecting amyloid-β brain balance and the putative role of PPARs

**DOI:** 10.3389/fnagi.2014.00176

**Published:** 2014-07-28

**Authors:** Juan M. Zolezzi, Sussy Bastías-Candia, Manuel J. Santos, Nibaldo C. Inestrosa

**Affiliations:** ^1^Laboratorio de Biología Celular y Molecular, Departamento de Biología, Facultad de Ciencias, Universidad de TarapacáArica, Chile; ^2^Departamento de Biología Celular y Molecular, Facultad de Ciencias Biológicas, Pontificia Universidad Católica de ChileSantiago, Chile; ^3^Centro de Envejecimiento y Regeneración (CARE), Departamento de Biología Celular y Molecular, Facultad de Ciencias Biológicas, Pontificia Universidad Católica de ChileSantiago, Chile; ^4^Centre for Healthy Brain Ageing, School of Psychiatry, Faculty of Medicine, University of New South WalesSydney, NSW, Australia; ^5^Centro de Excelencia en Biomedicina de Magallanes (CEBIMA), Universidad de MagallanesPunta Arenas, Chile

**Keywords:** brain homeostasis, blood-brain barrier, Aβ balance, systemic Aβ clearance, neurodegenerative disorders, nuclear receptors

## Abstract

Alzheimer’s disease (AD) is the most common form of age-related dementia. With the expected aging of the human population, the estimated morbidity of AD suggests a critical upcoming health problem. Several lines of research are focused on understanding AD pathophysiology, and although the etiology of the disease remains a matter of intense debate, increased brain levels of amyloid-β (Aβ) appear to be a critical event in triggering a wide range of molecular alterations leading to AD. It has become evident in recent years that an altered balance between production and clearance is responsible for the accumulation of brain Aβ. Moreover, Aβ clearance is a complex event that involves more than neurons and microglia. The status of the blood-brain barrier (BBB) and choroid plexus, along with hepatic functionality, should be considered when Aβ balance is addressed. Furthermore, it has been proposed that exposure to sub-toxic concentrations of metals, such as copper, could both directly affect these secondary structures and act as a seeding or nucleation core that facilitates Aβ aggregation. Recently, we have addressed peroxisomal proliferator-activated receptors (PPARs)-related mechanisms, including the direct modulation of mitochondrial dynamics through the PPARγ-coactivator-1α (PGC-1α) axis and the crosstalk with critical aging- and neurodegenerative-related cellular pathways. In the present review, we revise the current knowledge regarding the molecular aspects of Aβ production and clearance and provide a physiological context that gives a more complete view of this issue. Additionally, we consider the different structures involved in AD-altered Aβ brain balance, which could be directly or indirectly affected by a nuclear receptor (NR)/PPAR-related mechanism.

## Introduction

**Figure 1:**
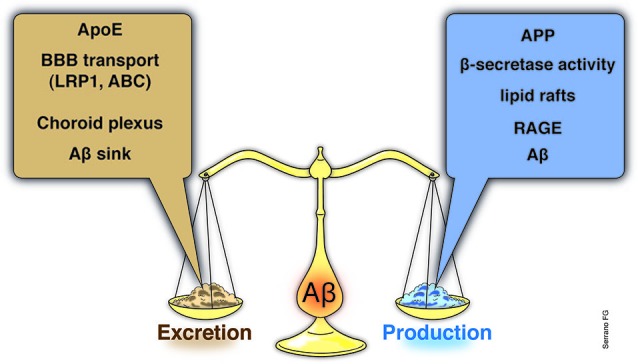
**Aβ brain balance, a systemic event.** Although the link between Aβ and AD has been known from decades, the importance of Aβ balance, as the result of clearance mechanisms along with brain Aβ production and influx events, has become important only recently. Moreover, the link between the Aβ brain levels and the involvement of brain adjacent tissues, such as the blood-brain barrier (BBB) or the ChP, as well as, with systemic alterations have been emerged as an interesting matter to examine. Indeed, recent studies have explored the potentialities of systemic interventions in order to reduce Aβ brain levels. Several studies have demonstrated that ApoE levels, the main Aβ chaperone within the brain, is a key element of Aβ brain removal and along with the BBB ApoE-related transporters account for almost the total Aβ brain clearance. Additional structures, such as the ChP, has also been demonstrated to play a key role in the Aβ removal from the brain to the CSF and to blood. At the basis of the Aβ brain clearance, emerge an Aβ sink established by the systemic excretion of the Aβ, a process carried out mainly by the liver and in less proportion by the kidneys. Whether normal or abnormal levels of Aβ production (increased APP or BACE expression, in the lipid rafts) the Aβ sink in the final Aβ brain balance is clearly critical. If an impaired systemic Aβ excretion due to failure of the liver or kidney, compromise the chances to properly reduce the blood Aβ charge, and additional elements, such as the RAGE, might start to act and inducing Aβ influx to the brain, starting or aggravates the Aβ accumulation. BBB, blood-brain barrier; ChP, Choroid plexus; ApoE, apolipoprotein E; APP, amyloid precursor protein; BACE, β-site APP cleaving enzyme; RAGE, receptor for advanced glycation end products.

During recent decades, it has become evident that the efficiency of an organism’s homeostatic mechanisms is closely related to its lifespan, suggesting that aging implies the alteration/modification of several cellular processes necessary to sustain homeostasis (Buga et al., 2011; Popa-Wagner et al., 2011; Basha and Poojary, 2014; Ureshino et al., 2014). Interestingly, aging is recognized as the primary risk factor associated with some chronic degenerative diseases, such as cancer, and/or some neurodegenerative disorders, such as Alzheimer’s (AD) or Parkinson’s disease (Zlokovic et al., 2010). Moreover, recent published works strongly suggest that the clearance of amyloid-β (Aβ), a key peptide in AD, and the alteration of this mechanism could be closely related to different stages of the disease, e.g., the establishment and/or progression of AD (Figure 1; Cramer et al., 2012). A genetic component has been described for this disease (familial form); however, it is important to note that genetic-based cases usually account for a limited or reduced number of total cases.

In the present review, we approach the Aβ clearance problem from different perspectives, including the molecular basis of Aβ imbalance, systemic considerations that favor or impair Aβ final excretion, and a wider view of how different tissues should interplay to ensure Aβ balance, thus preventing the development of pathologic processes. In the same manner, based on our experience, we discuss the perspectives regarding nuclear receptors (NRs) stimulation, particularly peroxisome proliferator-activated receptors (PPARs) and some of the cellular signaling pathways that could be behind the effects observed for this family of NRs.

## AD overview

AD is an age-associated neurodegenerative disorder characterized by progressive memory loss and cognitive impairment, and it is related to selective neuronal death in memory and learning brain areas, which eventually leads to patient disability and ultimately death (Braak and Braak, 1991; Morgan et al., 2007; Salmon and Bondi, 2009; Savva et al., 2009; Ballard et al., 2011; Serrano-Pozo et al., 2011; Godoy et al., 2014). Although many efforts are committed to AD research, this disease represents a prevalent neurodegenerative disorder that has become a serious public health concern due to the aging of the world population (Lutz et al., 2008). Clinically, AD precipitates a gradual neurodegeneration affecting the short-term memory at the beginning of the disease, followed by long-term memory loss (Braak and Braak, 1991; Gómez-Isla et al., 1997; Perl, 2010). Brain atrophy and gradual loss of neurons, mainly in the hippocampus, frontal cortex, and limbic areas, together with the extracellular accumulation of Aβ plaques and the intra-neuronal formation of neurofibrillary tangles (NFT), are pathological hallmarks of the disease (Salmon and Bondi, 2009; Perl, 2010; Manji et al., 2012). Whether in the familial or sporadic form, increased levels of Aβ have been described as the starting point of the pathological changes observed in AD (Selkoe, 2001; Karran et al., 2011). Aβ aggregates are often surrounded by dystrophic neurites and reactive glial cells, and Aβ peptide has been described as the major neurotoxic agent causing these alterations (Li et al., 2010). Moreover, recent evidence clearly supports the hypothesis that Aβ oligomers are a key factor in synaptic impairment and the spatial memory decline associated with neuronal dysfunction (Lacor et al., 2004; Haass and Selkoe, 2007; Cerpa et al., 2008; Dinamarca et al., 2012), including the synaptic failure associated with the loss of synaptic proteins that contributes to the progression of the disease (Scheff et al., 2007; Mucke and Selkoe, 2012; Borlikova et al., 2013). Additionally, it have been consistently demonstrated that Aβ also affects energy homeostasis mainly because an altered insulin signaling and due to Aβ-induced mitochondrial dysfunction (Abramov et al., 2004; Paula-Lima et al., 2011; Popa-Wagner et al., 2013), suggesting a severe cellular compromise which leads to general failure of the cellular machinery.

These neurodegenerative pathological changes of AD ultimately reflect the damage of the neuronal network due to altered synaptic structure and synaptic functionality (Perl, 2010; Sheng et al., 2012; Godoy et al., 2014). Pathologic modifications of the presynaptic neurotransmitter-releasing machinery and/or altered expression of specific postsynaptic proteins, such as the postsynaptic density protein-95 (PSD-95), are at the basis of the synaptic impairment observed in AD (Sheng et al., 2012; Südhof, 2012, 2013). Importantly, although neuronal network damage occurs across the entire brain, the hippocampus, which is associated with memory and cognition, is one of the most critically involved regions (Oliva et al., 2013; Shaerzadeh et al., 2014).

Regrettably, although AD was described more than a century ago and important progress has been made in the understanding of this disease, effective AD treatments remain elusive because there are no disease-modifying therapies that can slow or definitively stop the progression of the neurodegenerative process (Langbaum et al., 2013). From the initial cholinergic hypothesis to the actual *tau* and amyloid hypotheses, research has confirmed several aspects of AD-involved molecular pathways; however, no satisfactory mechanisms have been revealed to enable an effective intervention against this disorder. Recently, an increasing body of evidence has directed attention toward the mechanisms involved with Aβ balance, namely the Aβ production/excretion rate (Cramer et al., 2012; LaFerla, 2012; Fitz et al., 2013; LaClair et al., 2013; Landreth et al., 2013; Price et al., 2013; Tesseur et al., 2013; Veeraraghavalu et al., 2013; Zolezzi and Inestrosa, 2014).

## Molecular basis of Aβ biology: physiological and pathological considerations

**Figure 2:**
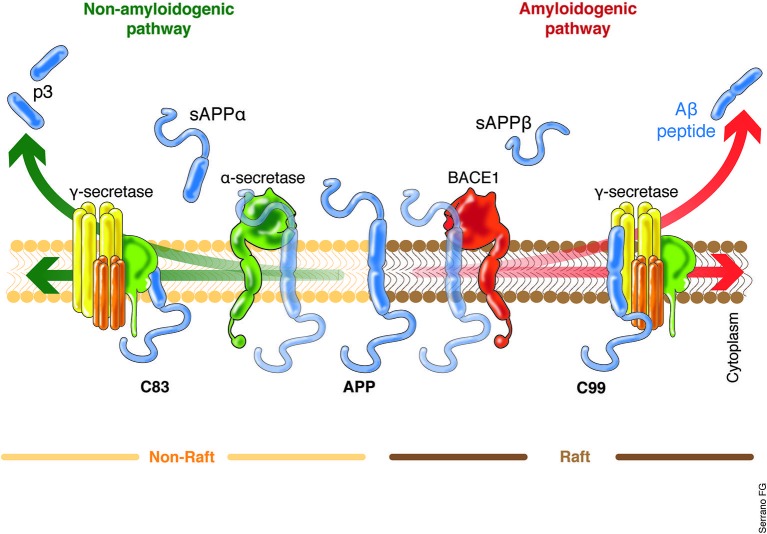
**APP processing, critical cellular choice.** The main source of Aβ production within the brain are the neurons. Two proteolytic processing pathways of APP have been described with two clear outputs. The non-amyloidogenic pathway will lead to the final release of the p3 and sAPPα, a small peptide with still poorly understood cell function. The cleaving enzymes which act to produce the sAPPα are the α- and γ-secretase. On the other hand, the activity of the β- and γ-secretase leads to the formation of the sAPPβ and the Aβ, the main neurotoxic agent described in AD. The role of the BACE is out of question and it is considered the Aβ production rate limiting enzyme. Interestingly, the recent work of Singh et al. (2013) clearly indicates that external factors might influence the expression levels of BACE, suggesting the potential up-regulation of the amyloidogenic processing of the APP. In the same context, it have been recently proposed that the APP amyloidogenic processing machinery is located in the lipid rafts rich in cholesterol. The increased lipid content within the cells, for example, as a result of increased systemic lipids levels, might also influence which APP processing machinery will be prompted to act. sAPPα/β, soluble APP fragment α/β; p3, 3-KDa peptide; BACE, β-site APP cleaving enzyme.

Aβ is a 37–49 peptide generated from the post-translational amyloidogenic processing of the amyloid precursor protein (APP), a transmembrane protein that is present in several cell types, including neurons. The precise function of the APP remains not fully understood, although nervous system nerve differentiation during development and both signaling and cell adhesion have been related to this protein (Turner et al., 2003; Priller et al., 2006; Zheng and Koo, 2006). APP possess a highly complex processing machinery, including three site-specific cleaving enzymes termed α-, β-, and γ-secretase, the differential action of which leads to the non-amyloidogenic or amyloidogenic processing of APP (Figure 2). The coordinated processing of α- and γ-secretase leads to the formation of soluble APP-α (sAPPα) fragments, while the action of β- and γ-secretase causes the release of sAPPβ and the neurotoxic Aβ (Grimm et al., 2013; Yan and Vassar, 2014). β-secretase, also known as β-site APP cleaving enzyme (BACE1 and 2), is considered to be the Aβ production rate limiting enzyme, and BACE-directed therapy is currently one of the aims of several research projects (Grimm et al., 2013; Buggia-Prévot et al., 2014; Yan and Vassar, 2014). Similarly, mutations in any of the γ-secretase subunits, particularly presenilin (PSEN1 and 2), have been proven to induce the aberrant processing of the APP, causing an increase in Aβ levels and favoring AD early onset (Bekris et al., 2011; Benitez et al., 2013; Larner, 2013). Increasing interest in β- and γ-secretase clustering has emerged in various investigations, which indicate that this event is favored in cholesterol-rich domains of the plasma membrane, termed lipid rafts (Kapoor et al., 2010; Marquer et al., 2011). Some authors have proposed that lipid rafts would be appropriate targets of potential therapeutic interventions against AD (Ben Halima and Rajendran, 2011).

The significance of APP processing and the importance of BACE results are evident from several studies focused on understanding the cognitive decline and the memory impairment observed in patients with chromosome 21 trisomy, where APP and BACE genes are encrypted (Mok et al., 2013). The critical role of BACE as an Aβ-level modulator is no longer debated, and recent work has suggested the importance of understanding how BACE polymorphisms determine not only Down’s syndrome AD onset but also sporadic AD cases (Zhou et al., 2010; Mok et al., 2013; Natunen et al., 2013). Moreover, the recent work of Singh et al. (2013), which demonstrates that sub-toxic plasma concentrations of copper may influence the expression of BACE1, highlights the importance of non-evident or non-clinical events that could be at the basis of some of the pathological changes that will ultimately lead to AD onset.

It is important to note that the deficient expression of genes related to the non-amyloidogenic processing of APP, such as ADAM 9, 10, and/or 17, which have demonstrated α-secretase activity, should also be addressed. These genes are related to increased Aβ levels as a consequence of the increased amyloidogenic processing of APP (Bekris et al., 2011).

Due to the complexity of APP processing and the genes involved in this process (from the APP itself to the genes coding for each of the subunits necessary for the APP post-translational modification), the study of the genetic variations, such as polymorphisms or single nucleotide polymorphisms (SNPs), is mandatory to correctly evaluate each patient and to develop directed therapies that are not based on underestimated genetic conditions. In the same way, we believe that a deep understanding of this matter should enable the development of new *in vitro*/*in vivo* models of AD that are necessary to evaluate new therapeutic strategies.

## Brain Aβ levels in the interstitial fluid (ISF), cerebrospinal fluid (CSF) and blood

Current knowledge indicates that Aβ begins to accumulate outside the cell, within the interstitial fluid (ISF), where its aggregation might be facilitated due to an altered microenvironment leading ultimately to the formation of senile plaques (Näslund et al., 2000; Karran et al., 2011; Li et al., 2012). It was initially believed that plaques were responsible for neuronal damage and the concomitant cognitive impairment, but the poor correlation between plaque burden and cognitive compromise prompted researchers to question the role of the plaque in AD ethiology (Lesné et al., 2013). Today, it is widely accepted that it is not the plaque but instead the Aβ oligomers levels that are the basis of neuronal damage (LaFerla et al., 2007; Lesné et al., 2013). Although the following remains controversial, several authors have proposed that the intracellular accumulation of Aβ could account for the initial synapse and neurite damage registered during the first stages of the disease (LaFerla et al., 2007; Gouras et al., 2010; Zheng et al., 2012). The mechanisms regarding intracellular Aβ accumulation have been proposed to be related to endogenous cellular aspects, such as the intracellular APP export and cleavage, which can occur wherever APP encounters the necessary enzymatic machinery (LaFerla et al., 2007; Gouras et al., 2010; Jiang et al., 2014), and to an altered neuronal catabolism of Aβ (Nilsson and Saido, 2014). Regarding the first, it is quite important to note that APP have been encountered in different cellular compartments, such as Golgi, endoplasmic reticulum (ER), endosomal, lysosomal, and mitochondrial membranes (Mizuguchi et al., 1992; Xu et al., 1995; Kinoshita et al., 2003; Zheng et al., 2012). On the other hand, autophagy has been recognized as a critical cellular process which impairment results determinant for increased intraneuronal Aβ levels. Alterations in Rab GTPases family members as well as altered activity of lysosomal enzymes, such as cathepsins, are part of the basic cellular mechanism to deal with Aβ (Nixon et al., 2001; Nilsson and Saido, 2014). As mentioned above, it has been proposed that when this systems fails, it will allow the rise of intracellular Aβ levels leading to the accumulation and aggregation of Aβ within the cells and, ultimately to cell death (Li et al., 2012; Nilsson and Saido, 2014). Additionally, Aβ reuptake has been described and is of the most interest in the context of the high affinity between Aβ and the α7 nicotinic acetylcholine receptor (LaFerla et al., 2007; Inestrosa et al., 2013), a situation that leads to the internalization of the receptor/Aβ complex and increasing intracellular Aβ levels.

Whether of an extracellular or intracellular origin, the Aβ must finally be removed from brain parenchyma in order to prevent its accumulation and aggregation (Karran et al., 2011). At this point, the activity of glial cells is fundamental not only due to the phagocytic activity that they exert against Aβ (Guo et al., 2004; LaFerla, 2012; Zhu et al., 2012), but because they are the primary source of apolipoprotein E (ApoE), which is the main chaperone of Aβ within the central nervous system (CNS; LaDu et al., 2000). To date, three isoforms of ApoE have been described (ɛ2, ɛ3, and ɛ4), and the ApoEɛ4 variant is considered to be one of the most relevant risk factors for AD (Corder et al., 1993; Zhu et al., 2012; Tai et al., 2014). Additionally, ApoJ, transthyretin and α2-macroglobulin (α2M) have been described as secondary chaperones and are considered to play a role in Aβ brain efflux (Deane et al., 2008). Considering the relevance of ApoE, it is clear that the expression of this chaperone could strongly influence the rate of Aβ brain removal. Several authors have proposed ApoE as a primary target for future AD therapies (Cramer et al., 2012; Frieden and Garai, 2012; Lane et al., 2012).

Additionally, Aβ could undergo enzymatic degradation via neprilysin, the main soluble Aβ degrading enzyme, the expression of which has been reported as decreased in brains of several murine models of AD and in *in vitro* models (Tampellini et al., 2011; Grimm et al., 2013). Moreover, several authors have suggested a direct link between the APP process and neprilysin regulation in a type of feedback regulatory mechanism that is directed by the APP intracellular domain released during APP cleavage (Vásquez et al., 2009; Grimm et al., 2013). However, neprilysin is only able to degrade soluble forms of Aβ; thus, once the insoluble Aβ forms, such as fibrils, are present, the role of glial cells and matrix metalloporteases, such as MMP-1, -2 and -9, is fundamental and, as has been demonstrated systematically, alterations in glial response as well as an altered activity of MMPs could be well related to neurodegeneration and AD (Mroczko et al., 2013; Table 1). In addition to the enzymatic removal of Aβ, efflux to the blood across the blood-brain barrier (BBB) and via drainage from the CSF complements the brain Aβ clearance system (Deane et al., 2008).

**Table 1:** **Aβ levels critical control points**.

**Degradation**	
Intracellular	Autophagy (Lysozymes: cathepsins)
Extracellular	
monomers	Neprilysin
insoluble forms	Matrix Metalloproteases (MMPs: 1, 2, 9)
**Transport**	
ApoE	Aβ chaperone
ABC	Transporters family related to ApoE movilization
LRP1	Main ApoE receptor
sLRP1	plasmatic soluble fragment of LRP1, Aβ chaperone

*ApoE, apolipoprotein E; ABC, ATP binding cassette; LRP1, low density lipoprotein related receptor protein 1; sLRP1, soluble LRP1*.

### BBB and choroid plexus (ChP) Aβ transporters

Aβ transport across the BBB is the main pathway in maintaining appropriate brain Aβ levels. While this primary mechanism directly exports Aβ from the brain ISF to the blood, a secondary pathway involving ChP/CSF bulk flow and CSF/blood Aβ exchange at the Virchow-Robin space also contributes to brain Aβ balance (Deane et al., 2008). Due to its electrochemical nature, Aβ requires specialized carriers to cross the BBB and ChP barriers (Zlokovic, 2010; Zolezzi and Inestrosa, 2013). Importantly, the carriers present at each barrier are the same (Pascale et al., 2011).

The low-density lipoprotein receptor-related protein (LRP1 and 2) and the ATP binding cassette (ABCB1, C1, G2, and G4) are the two main families of transporters related to brain Aβ efflux (Bell et al., 2007; Bell and Zlokovic, 2009; Jaeger et al., 2009; Cramer et al., 2012; Kanekiyo et al., 2012). Although both pathways play an important role in Aβ clearance, several studies suggest that BBB alteration is not only a consequence of the AD neurodegenerative process but could be the basis of these changes (Zlokovic, 2010, 2011; Erickson and Banks, 2013; Zolezzi and Inestrosa, 2013). In the same manner, any genetic variation of such transporters could have an enormous impact on the establishment and progression of AD (Erickson and Banks, 2013; Zolezzi and Inestrosa, 2013).

**Figure 3:**
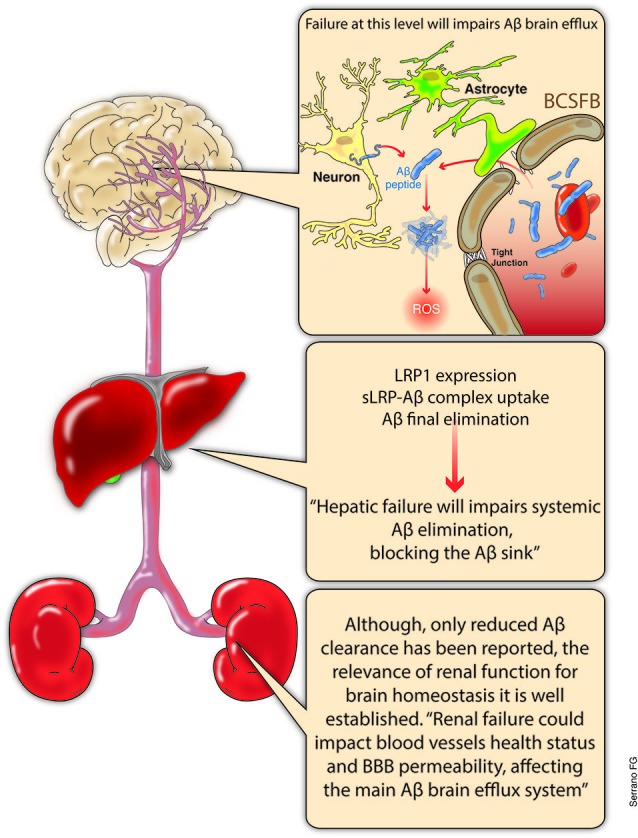
**Aβ balance, systemic overview**. The main discussion regarding Aβ clearance has been centered at the brain level. Increased production and decreased removal from the brain certainly constitutes a highly relevant issue. The relevance of the BBB integrity or the Aβ excretion through the ChP are now recognized as key elements regarding Aβ brain levels. However, a growing body of evidence suggest the critical role of systemic final excretion of Aβ in AD. In this regard, expression levels of LRP 1 within the liver and hepatocyte are critical for the appropriate liver excretion of Aβ, which could account for up to the 60% of the total systemic Aβ clearance. On the other hand, even when not fully understood, kidneys not only play an important role in systemic Aβ clearance, but the precise renal function might account for blood vessels health and appropriate blood pressure levels which could influence the BBB integrity and its functionality. LRP, low density lipoprotein-related receptor protein; sLRP-Aβ, soluble LRP bond to Aβ; BCSFB, brain-cerebrospinal fluid barrier; ChP, choroid plexus.

It is important to note that the main Aβ chaperone in the plasma is the soluble form of the LRP and in the CSF is the lipocalin-type prostaglandin D synthase β-trace (Deane et al., 2008; Sagare et al., 2011). This situation is most relevant for final Aβ elimination, a process that primarily occurs in the liver (Ghiso et al., 2004; Tamaki et al., 2006; Sagare et al., 2012), and to a lesser extent, in the kidneys (Ghersi-Egea et al., 1996; Sagare et al., 2007; Figure 3).

## AD and the systemic regulation of Aβ levels: the role of the liver and kidneys

As previously mentioned, the liver is the most important place for final Aβ removal, where the binding of liver LRP1 to the Aβ and the posterior elimination generates a sink that ensures continuous Aβ elimination (Sagare et al., 2012). The works of Tamaki et al. (2007) and Ito et al. (2010) provided evidence that the blockade of LRP1-Aβ binding in the liver causes an increase of plasmatic Aβ levels, which could be related with increases in brain Aβ levels. It has been demonstrated that an increase in the plasma levels of Aβ could induce Aβ influx into the brain through a specific BBB transporter, the receptor for advanced glycation end products (RAGE; Deane et al., 2012; Sagare et al., 2012), leading to accumulation and aggregation in the brain, with subsequent damage to the neuronal network. Therefore, the appropriate expression level of liver LRP and the health status of hepatocytes are of great relevance in regulating systemic Aβ levels and in avoiding dangerous increases of this neurotoxic agent (Sagare et al., 2012).

On the other hand, although the renal excretion of sLRP and Aβ has been described, the relevance of this process has been poorly addressed (Sagare et al., 2007; Shea et al., 2014). However, as evidenced by several authors, vascular health, as a result of an appropriate renal function, plays a fundamental role in AD establishment and progression (Zlokovic, 2010, 2011; Erickson and Banks, 2013; Zolezzi and Inestrosa, 2013, 2014). Cerebral microinfarcts, microbleedings, elevated blood pressure, cardiac failure, and stroke are only some of the pathological conditions that reflect or could alter blood vessels (Zlokovic, 2010, 2011). Moreover, the relationship between the compromise of renal function and pathological changes in the brain has been demonstrated (Liu et al., 2008; Busch et al., 2012). However, there is a lack of knowledge regarding this issue, and it should be considered when a multisystemic approach to AD or other neurodegenerative disorders is pursued.

## Nuclear receptors (NRs): PPARs and their potential role in a multisystemic therapeutic strategy

NRs are a highly complex transcription factor superfamily that is fundamental for several cell processes. The main function of NRs has been related to both the extracellular and intracellular media (Olefsky, 2001). NRs play a critical role within cells, as indicated in several reports that correlated NR dysfunction with pathological conditions such as cancer, insulin resistance and infertility (Olefsky, 2001; Gronemeyer et al., 2004). As cell sensors, NRs interact with different cellular signaling pathways, such as Wnt, phosphoinositide 3-kinase (PI3K) and mitogen-activated protein kinases (MAPK), exerting gene expression regulation of a wide range of target genes (Mulholland et al., 2005; Fuenzalida et al., 2007; Inestrosa and Toledo, 2008).

NRs can be divided into two main categories: Type I, such as the androgen, estrogen, and progesterone receptors; and Type II, including the thyroid receptor, the retinoid X receptor (RXR) (homodimer), the vitamin D receptor, the retinoic acid receptor, the liver X receptor (LXR), and the PPARs (Olefsky, 2001; Mulholland et al., 2005; Zolezzi and Inestrosa, 2013, 2014). The main difference between types is their ability to form homodimers (Type I) or heterodimers with the RXR (Type II) (Mulholland et al., 2005).

Several studies have been conducted on the pharmacological potentialities of different NRs, including cancer research, neurodegenerative disorders, and acute brain injury, among others (Aleshin et al., 2013; Fu et al., 2014; Garattini et al., 2014). Among the NR superfamily, PPARs are the most studied ones (Aleshin et al., 2013).

To date, three different mammalian PPARs have been identified: PPARα, PPARβ/δ, and PPARγ (Neher et al., 2012). Although all PPARs have been described in both the adult and developing brain (Heneka and Landreth, 2007), PPARγ is the most studied isoform and has shown the most promising neuroprotective effects in different models of neurodegenerative disorders, such as AD (Inestrosa et al., 2005, 2013; Santos et al., 2005; Toledo and Inestrosa, 2010; Chen et al., 2012; Neher et al., 2012). A common feature of PPARs is that part of it activity is mediated by the direct binding to DNA, specifically to the peroxisome proliferators-response elements (PPREs), a DNA consensus sequence (AGGTCA-N-AGGTCA) localized mainly at the promoter region of PPARs-genes (Heinäniemi et al., 2007). However, as mentioned above, when potential PPARs target genes are evaluated, the RXR target genes must also be considered. Several genes have been linked to the different PPARs, including some Apo-family of lipid transporters; other nuclear receptors, such as LXR; the UCP-3 (energy metabolism); among others (Kanehisa and Goto, 2000; Heinäniemi et al., 2007; Kanehisa et al., 2014). Interestingly, some authors have demonstrated that among the PPAR target genes might also be present some key components of relevant cellular signaling pathways, such as Wnt (Toledo and Inestrosa, 2010) and mTOR (Hagland et al., 2013), among others.

Although PPARs were identified long ago, the recent work of Cramer et al. (2012) has directed attention to this nuclear receptor subgroup as a key target for Aβ clearance in AD therapy. Indeed, prior to Cramer’s work, several authors have already stated the relevant role of PPARs in the brain Aβ-clearance (Camacho et al., 2004; Kalinin et al., 2009; Escribano et al., 2010; Espuny-Camacho et al., 2010). Our laboratory and others, have been working with PPARs for many years, and we have systematically described the benefits of PPARs activation in several *in vitro* and *in vivo* models of AD (Fuentealba et al., 2004; Inestrosa et al., 2005, 2012; Fuenzalida et al., 2007; Nenov et al., 2014). Moreover, recent works suggest an interesting role for PPARs in mitochondrial dysfunction protection and functionality (Zolezzi et al., 2013a,b), which could be part of a series of PPAR-triggered mechanisms at the foundation of the benefits observed against AD.

However, it is important to note, that the vast majority of information regarding PPARs benefits against neurodegenerative disorders, such as AD, have arose from *in vitro* and *in vivo* studies based on different animal models. Moreover, some clinical trials have been conducted, with dissimilar results, and others are actually under development (Ryan, 2014). On this regard, several questions remains regarding PPARs mechanisms of action.

### PPARs and the BBB

Among the Aβ neurotoxic mechanisms, oxidative stress and mitochondrial damage are two of the most cited effects of Aβ exposure. Several authors have suggested that the perivascular accumulation of Aβ damages the BBB, leading to microbleedings, inflammatory reactions, and subsequent damage to the neuronal network (Zlokovic, 2010; Popa-Wagner et al., 2013; Zolezzi and Inestrosa, 2013). On this regard, several authors have demonstrated the role of PPARs as an endothelial protective agents (Zhou et al., 2008; Bae et al., 2010; Kröller-Schön et al., 2013; Zarzuelo et al., 2013; d’Uscio et al., 2014; Hawkes et al., 2014). Recently, it has been demonstrated that PPARs are able to protect endothelial cells from oxidative damage, thus preventing vascular dysfunction, which could favor brain parenchyma alterations (d’Uscio et al., 2012; Papadopoulos et al., 2013). Based on current knowledge and on our own work, we have proposed that PPAR activation, through natural or synthetic ligands, could protect and recover BBB integrity and functionality by increasing cell antioxidant capacity and improving energy metabolism, leading to the increased expression of specific transporters that could influence the Aβ-clearance rate (Nicolakakis et al., 2008; Zolezzi and Inestrosa, 2013; Zolezzi et al., 2013b; Hawkes et al., 2014). Energy metabolism is vital for both, neurons and the BBB, primarily because the preservation of the ion gradients (in the case of neurons) and the traffic across the BBB requires large amounts of energy (Abbott et al., 2010; Liebner and Plate, 2010; Popa-Wagner et al., 2013).

**Figure 4:**
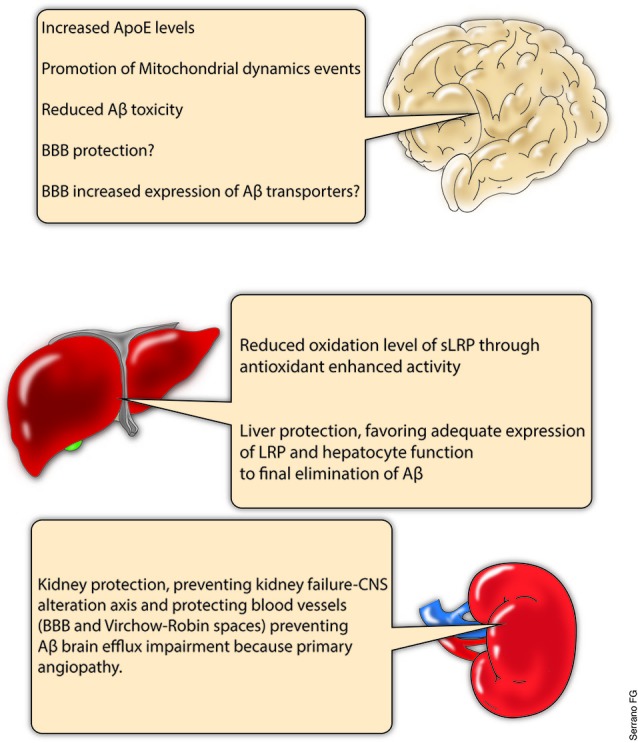
**PPARs, potential for systemic Aβ clearance.** PPARs are a complex subfamily of NRs. Several PPARs agonists have been studied under different physiological and pathological conditions, and numerous effects have been reported for this group of drugs in several organs. Central nervous system (CNS), liver and kidneys are some of the tissues which have demonstrated to respond to PPAR agonist treatments. In this regard, the present scheme summarizes part of the current knowledge relative to PPARs agonists and the potential that they might exert in different organs regarding the Aβ systemic clearance. Of course, much research is needed in order to properly address the importance of PPARs as therapeutic agents, but the approach presented here suggest the study of new therapeutic strategies including additional intervention levels.

Although the main effects resulting from PPAR stimulation have been related to microglial and astrocytic activation as the key events that allow brain Aβ clearance (Mandrekar-Colucci et al., 2012; Yamanaka et al., 2012), additional mechanisms, such as the PPARγ-LXR-mediated increased expression of ApoE (Cramer et al., 2012; Mandrekar-Colucci et al., 2012) along with the increased expression of ApoE-Aβ carriers (the ABC family of transporters), indicate a close relationship between these mechanisms and the foundational role of Aβ trafficking across the BBB that can properly explain the benefits observed after PPAR stimulation in several models of AD (Mysiorek et al., 2009; Cramer et al., 2012; Hoque et al., 2012; Figure 4). Importantly, although different authors recognize the relevance of the BBB traffic system, only a small proportion of research has focused on the disease-related expression variations of BBB transporters. Less is known regarding the disease-induced modification of transporters at the ChP, indicating that this is an enormous field to investigate.

### PPARs and the systemic clearance of Aβ

As previously indicated, the main plasmatic chaperone protein of Aβ is sLRP (Tamaki et al., 2006; Sagare et al., 2012). Interestingly, sLRP has been reported to be in an oxidized state (which reduces the affinity of sLRP for Aβ) in AD patients (Sagare et al., 2007). Several investigations suggest that the activation of PPARs can protect against oxidative damage (Hernanz et al., 2012). Additionally, PPARs have been demonstrated to protect the liver, thus preventing the impairment of systemic antioxidant production and the loss of intact hepatocytes with LRP surface expression, which enables the final excretion of Aβ (Iwaisako et al., 2012; Patterson et al., 2012; Figure 4).

Similarly, several authors have reported the protective activity of PPARs at the renal level. Renal fibrosis or necrosis after ischemic insults are two of the events that could influence renal functionality, thereby altering the clearance rate of Aβ in the kidneys (Fedorova et al., 2013; Li et al., 2013). Regrettably, there is little information regarding PPARs and kidneys and PPAR implication in AD or in other neurodegenerative disorders. However, it is possible that even when the Aβ clearance rate is not a determinant for a systemic Aβ balance, the role that kidneys play in blood pressure and/or the filtration of excretion products should have a great impact not only at the blood vessel level but also in the brain (Figure 4).

## Molecular basis of PPARs activity

The complexity of the response to PPAR stimulation arises from several cellular signaling pathways that have been described to be related to it. Interactions with several antioxidant and anti-inflammatory regulatory pathways, such as nuclear factor kappa-light-chain-enhancer of activated B cells (NF-κB), nuclear factor erythroid 2-related factor (NRF2), brain-derived neurotrophic factor (BDNF), and the Wnt/β-catenin pathway have been described (Zhang et al., 2011; Benito et al., 2012; Martín et al., 2012; Haskew-Layton et al., 2013; Benedetti et al., 2014). Additionally, it has been proposed that PPARγ can upregulate Bcl-2, which is an antiapoptotic protein and a Wnt target gene (Fuentealba et al., 2004; Fuenzalida et al., 2007). Over the last few years, it has been further proposed that the administration of PPAR agonists induces additional effects regarding neuronal functionality, including neurite outgrowth, and has a direct effect on mitochondrial fusion-fission dynamics (Feinstein et al., 2005; Chiang et al., 2012; Cho et al., 2013; Quintanilla et al., 2013; Zolezzi and Inestrosa, 2013; Zolezzi et al., 2013a).

Recently, we found that PPAR agonists are also able to induce mitochondrial dynamic events through PGC-1α. This process will prevent the mitochondrial dysfunction caused by oxidative insults, suggesting that cell metabolism is protected and that mitochondrial biogenesis should increase (Feinstein et al., 2005; Chiang et al., 2012; Pipatpiboon et al., 2012; Popa-Wagner et al., 2013; Zolezzi and Inestrosa, 2013; Zolezzi et al., 2013a). This latter finding is highly relevant considering that mitochondrial dynamics have recently been described as a critical mechanism associated with mitochondrial and cellular fate after critical insults (Manji et al., 2012). Such dynamics help sustain cell metabolism, and successive fusion-fission cycles enable the elimination of dysfunctional organelles and the repair of mitochondrial DNA that could be damaged after a toxic challenge (Haemmerle et al., 2011; Hondares et al., 2011; Silva et al., 2013; Zolezzi et al., 2013a). Moreover, as noted for antioxidant activity, the mitochondrial effects derived from PPAR activation could also be related to several cell signaling pathways such as Wnt (Silva-Alvarez et al., 2013). Recently, the activity of PPARs has also been proposed to be related to sirtuins (SIRT; Wang et al., 2013; Yang et al., 2013; Godoy et al., 2014), thus opening a new area for research and increasing the complexity of the molecular mechanisms involved with cellular PPAR response.

## Final considerations

Although published several years ago, the work of Cramer et al. (2012) clearly positioned Aβ clearance-related mechanisms as very promising candidates for future AD therapies. Moreover, their work prompted several authors to replicate or test old and new NR agonists to assess their effectiveness against Aβ accumulation. However, integrated studies that include systemic Aβ clearance and the effectiveness of systemic AD therapies are scarce. Our recommendation is that AD should be approached not only as a CNS issue but also from a multi-systemic perspective to accurately establish and define directed therapeutic interventions.

Indeed, the effects described by Cramer et al. (2012) and others partly involve the PPARs and suggest that PPARs should be considered as putative AD drugs. However, several questions have emerged regarding Cramer’s work which have highlighted the poor correlation of the benefits observed from bexarotene administration and the pathological markers evaluated by these researchers. Considering our experience on the subject, we believe that part of the controversy generated by Cramer’s work is due to a poor consideration of the mechanism behind PPAR stimulation. Thus, we propose a wider view of the Aβ clearance problem and the main key elements related to efficient Aβ elimination. Moreover, it is possible that different intervention points at which PPARs could influence the health of the systemic Aβ clearance machinery might be defined in the near future. As pointed previously, several clinical trials have attempted to transfer the *in vivo* results to real patients without success, but we think that there are still too many questions regarding NRs function (and particularly PPARs) to accurately estimate the effects of NR and PPAR stimulation.

## Author contributions

Each author participated actively in different manuscript preparation stages. Juan M. Zolezzi, Nibaldo C. Inestrosa, Sussy Bastías-Candia and Manuel J. Santos discussed and designed the present work. Juan M. Zolezzi and Nibaldo C. Inestrosa wrote and checked each subsection as well as the final version of the manuscript. Sussy Bastías-Candia and Manuel J. Santos wrote and corrected different subsection of the manuscript, as well as critically evaluated the final version of this work. Approval of the submitted final version was done by Nibaldo C. Inestrosa and Juan M. Zolezzi.

## Conflict of interest statement

The authors declare that the research was conducted in the absence of any commercial or financial relationships that could be construed as a potential conflict of interest.
